# Anatomical Characteristics of Extensor Hallucis Longus Tendon Variations and Its Clinical Implications: A Korean Cadaveric Study

**DOI:** 10.3390/ijerph19169833

**Published:** 2022-08-10

**Authors:** Jeong-Hyun Park, Yu-Jin Choi, Mijeong Lee, Digud Kim, Hyung-Wook Kwon, Kwang-Rak Park, Sa-Beom Park, Jaeho Cho

**Affiliations:** 1 Department of Anatomy & Cell Biology, School of Medicine, Kangwon National University, Chuncheon 24341, Korea; 2 Department of Anatomy, School of Medicine, Keimyung University, Daegu 42601, Korea; 3 Center of Biohealth Convergence and Open Sharing System, Hongik University, Seoul 04066, Korea; 4 Department of Orthopaedic Surgery, Chuncheon Sacred Heart Hospital, Hallym University, Chuncheon 24253, Korea

**Keywords:** clinical anatomy, anatomical variation, extensor hallucis longus, accessory tendon, cadaveric study

## Abstract

The purpose of this study is to ascertain the morphological characteristics of the extensor hallucis longus (EHL) tendon variation using larger-scale dissection of Korean cadavers and to classify the types of variation along with incidence. A total of 158 feet from adult formalin-fixed cadavers (50 males, 29 females) were dissected. The morphological characteristics and measurements of the EHL tendon variants were evaluated. Three types of the EHL tendon variation were classified, wherein the most common type was Type 2 (106 feet, 67.1%), Type 3 (3 feet, 1.9%) was the rarest type, and Type 1 without accessory tendon was found in 49 feet (31.0%). Type 2K (11 feet, 7%) and Type 3K (1 foot, 0.6%) were described as new subtypes. The present study suggests morphological characteristics of the EHL tendon variation in Korean populations and high morphological variability of the EHL tendon along with the possibility of differences according to race or ethnicity and gender. Furthermore, a newly updated classification complemented by new subtypes of variation will help foot and ankle surgeons in diagnosis and surgical planning with hallux problems.

## 1. Introduction

The extensor hallucis longus (EHL) muscle originates from the middle third of fibula and adjacent anterior surface of the interosseous membrane and is situated between and deep to the tibialis anterior (TA) muscle and the extensor digitorum longus (EDL) muscle. The EHL tendon extends through the inferior retinaculum and then inserts to the distal phalanx of the big toe (hallux). Especially, the EHL tendon reaches its insertion point at the base and dorsal surface of the distal phalanx of the big toe. Thus, the main action of EHL is to extend the big toe (hallux extensor) [1,2].

High variability in the anatomical morphology of the EHL has been reported and the most representative variation is the presence of the accessory tendon of the EHL. One or more accessory tendons of the EHL tendon have been well documented, particularly by frequency and insertion site of tendon [3,4,5,6,7,8,9], and it is presumed that there are regional and/or ethnic differences in the frequency and morphology of variation. Thus, an awareness of this variation has clinical significance to foot and ankle surgeons who always face hallux problems, such as the pathology caused by hallux deformity, and determine usefulness as donor for tendon graft [3,8,9,10].

Al-Saggaf first distinguished three different types with respect to insertion of the EHL tendon, as follows: single tendon, two tendons (main and medial accessory tendon), and three tendons (main, medial, and lateral accessory tendon) [3]. Later, Olewnik et al. proposed a systematic classification based on the number and the location of insertion of accessory tendons by adding new types [9]. Most recently, Zielinska et al. [11] has updated a new classification system that combines the both the Al-Saggaf [3] and Olewnik et al. [9] classification. In our routine dissection of Korean cadavers, however, the new patterns of the EHL tendon variation that were not described at all in the updated classification were observed.

Therefore, the current study was undertaken to ascertain the anatomical characteristics of the EHL tendon variation according to the number and the insertion site of accessory tendons using larger-scale dissection of Korean cadavers and to classify the types along with incidence.

## 2. Materials and Methods

All cadavers used in this study were donated through the donation program with consent for education and research in medical school. In addition, this study was approved by our institution of Institutional Ethics Committee (Institutional Review Board number: CHUNCHEON NON2021-002).

In this study, a total of 180 feet (90 adult cadavers) were dissected. Among those 180 feet, 158 feet (79 cadavers) were included for this study excluding 22 feet (11 cadavers) with abnormal signs of trauma or surgery, obvious deformities, pathologic lesions, and damage that render the investigation of morphology of structures difficult. Obvious deformities are those that do not have the foot shape in the normal range, but all cases wherein the morphology of structure can be investigated as deformity limited to general diseases (such as hallux valgus) were included.

**Figure 1:**
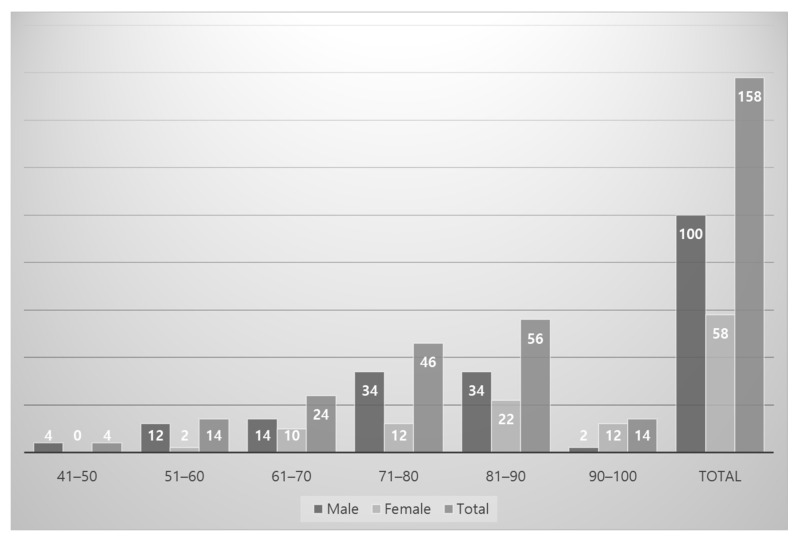
Gender and age distribution of Korean cadavers (*n* = 158 feet).

Of the 158 feet (79 cadavers) dissected from adult formalin-fixed cadavers, 100 feet (63%) were from males and 58 feet (37%) from females. The mean age of the donors at death was 79.9 (range, 44–100) years (Figure 1).

### 2.1. Dissection

The lower limb was fixed by a pedestal after formalin-fixed cadavers were placed in the supine position. Dissection started from the anterior aspect of the lower lime to the distal of foot dorsum. After the removal of the skin, the soft tissue was dissected layer by layer to expose the EHL muscle and tendon. Following this, the extensor retinaculum was removed to check the EHL tendon’s course from the ankle toward the hallux. The main and accessory tendons of EHL were carefully checked. Furthermore, the sites of tendon insertions were evaluated.

### 2.2. Assessment of Morphological Characteristics of the EHL Tendon Variations

Referring of the classification system by Zielinska et al. [11], authors classified types according to the number of EHL tendon and divided into subtypes according to the sites where the accessory tendons are distally inserted. If the new subtype found only in the current study was not included in the classification system by Zielinska et al. [11], it was classified as subtype ‘K’. Each independent researcher repeatedly investigated the morphology of the tendon insertion in duplicate. Identical consensus assessments from two researchers were adopted as data for each specimen.

The length of the main EHL tendon was measured from the point where it could be divided into the muscle belly and tendon to the distal insertion of tendon. Then, the width was measured at the midpoint of the main tendon. Furthermore, the length and width of accessory tendon were measured only in Type 2 (two tendons: main and one accessory tendon). For the length of the accessory tendon, the distance from the bifurcation of the main EHL tendon to the distal insertion point was measured. Next, the width of the accessory tendon was measured at the midpoint of the accessory tendon. An electronic digital caliper (Sincon Corporation, Seoul, Korea) with an accuracy of up to 0.1 mm was used for all measurements. For all measurements, two researchers each independently measured the width, and one researcher measured each once and the average was adopted as the measurement value.

### 2.3. Statistical Analysis

Intra- and interobserver reliability for all measurements were assessed by intraclass correlation coefficient (ICC). Data were analyzed using IBM SPSS Statistics version 23.0 for Windows (IBM Co., Armonk, NY, USA), and a *p*-value of less than 0.05 was considered statistically significant. The Fisher’s exact test was used to assess the distribution of types between gender and side. The mean comparison of length and width of tendons according to gender was analyzed by independent samples *t*-test, and the mean comparison between left and right was analyzed by paired *t*-test.

## 3. Results

The intraclass correlation coefficient for all measurements generated a result of 0.9 or higher. All measurements were higher than the accepted reliability (0.8) and were employed in the study.

The EHL tendon variation could be classified into three main types based on the number of tendons.

**Figure 2:**
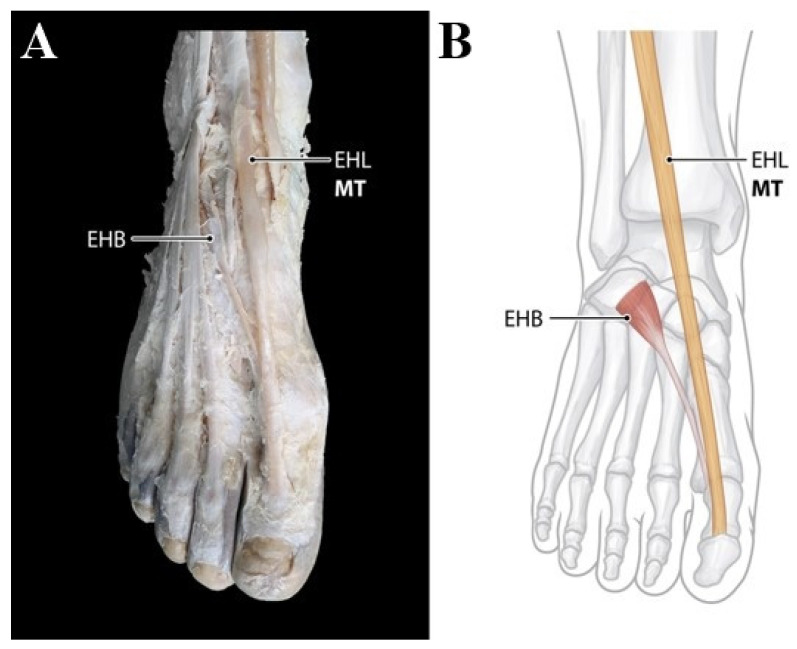
(**A**) Photo of Type 1 EHL tendon variation. The main single tendon inserts to the base of distal phalanx of the big toe. (**B**) Schematic drawing of Type 1 EHL tendon variation. EHB, extensor hallucis brevis tendon; EHL, extensor hallucis longus tendon; MT, main tendon.

Type 1 (single insertion) consists of single tendon. The main tendon only inserts to the base of distal phalanx of the big toe (hallux). This type was observed in 49 feet (31.0%) (Figure 2).

**Figure 3:**
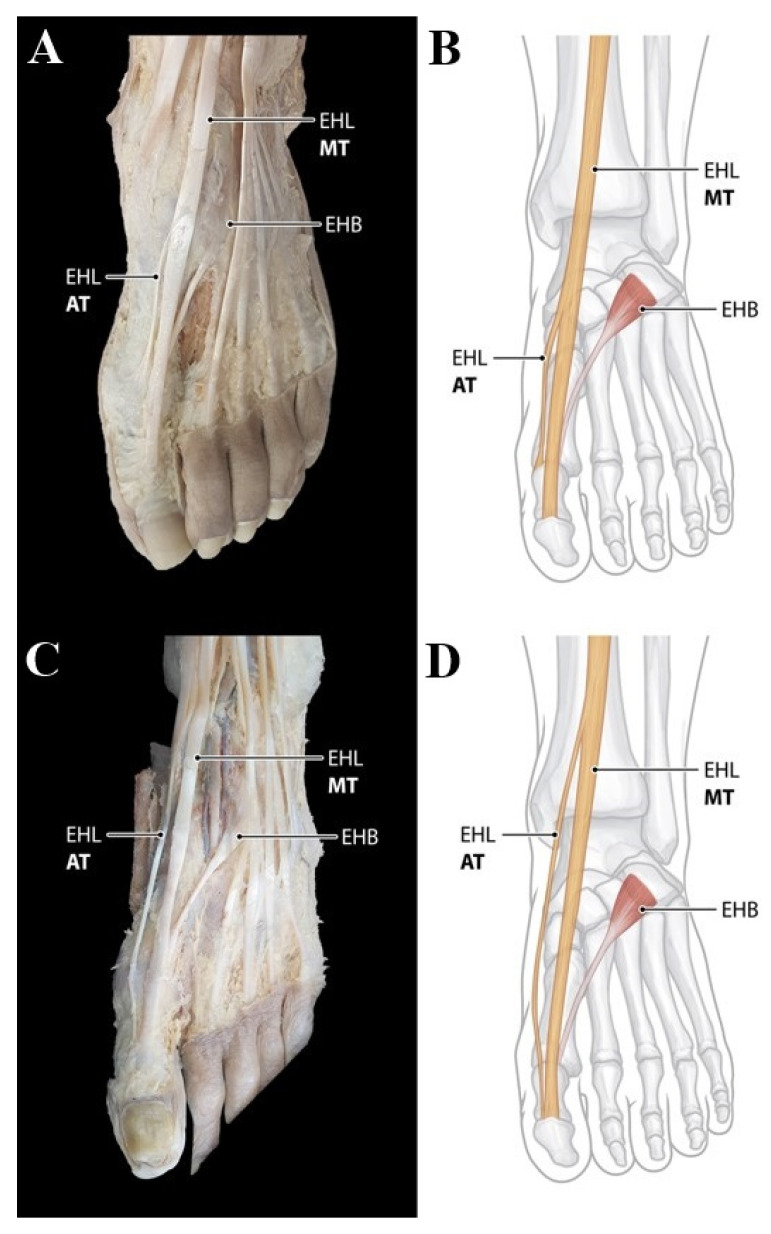
Type 2 EHL tendon variation. (**A**) Photo of Subtype 2A. The medial accessory tendon inserts to the dorsal aspect of the proximal phalanx base of the hallux. (**B**) Schematic drawing of Subtype 2A. (**C**) Photo of Subtype 2K. The medial accessory tendon inserts onto the first interphalangeal joint capsule. (**D**) Schematic drawing of Subtype 2K. AT, accessory tendon; EHB, extensor hallucis brevis tendon; EHL, extensor hallucis longus tendon; MT, main tendon.

Type 2 (double insertions) consists of two tendons, including the main tendon and one accessory tendon. The main tendon inserts as in Type 1; however, the following subtypes were divided according to the different insertion of the accessory tendons. Type 2 was observed in the 106 feet (67.1%). In Subtype 2A, described in the classification by Zielinska et al. observed in the 94 feet (59.5%), the medial accessory tendon inserts to the dorsal aspect of the proximal phalanx base of the hallux (Figure 3A,B). Subtype 2C of classification by Zielinska et al. was observed in only one foot (0.6%) and the lateral accessory tendon inserts to the dorsal aspect of the first metatarsal base. However, Subtype 2B, 2C, and 2E were not observed in our study. In addition, it is noteworthy that new subtype not mentioned in the classification by Zielinska et al. was founded in 11 feet (7.0%) of our study. In this subtype named ‘2K’, the medial accessory tendon inserts onto the first interphalangeal joint capsule (Figure 3C,D).

**Figure 4:**
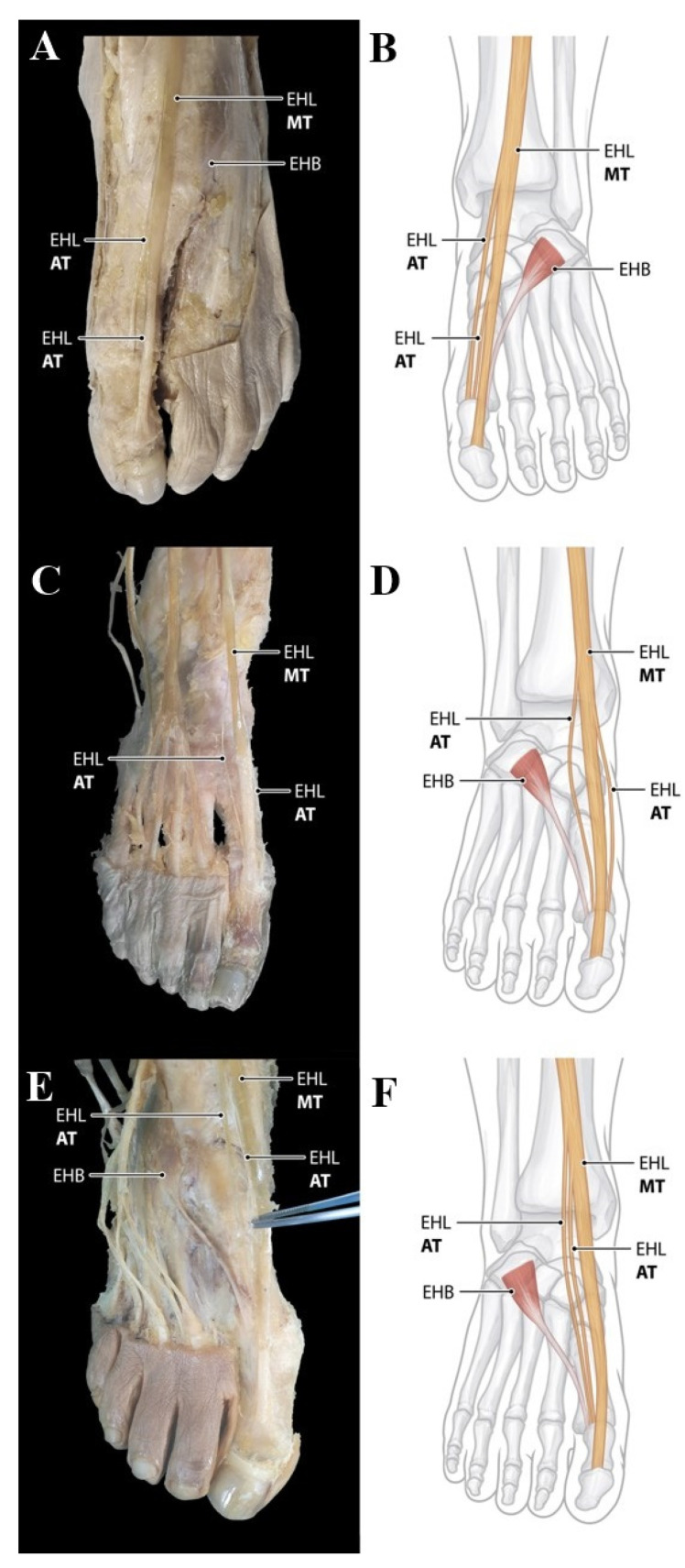
Type 3 EHL tendon variation. (**A**) Photo of Subtype 3B. Two accessory tendons arise from the medial side of the main tendon and insert into the capsule of first metatarsophalangeal joint. (**B**) Schematic drawing of Subtype 3B. (**C**) Photo of Subtype 3C. Two accessory tendons arise from the medial and lateral side of the main tendon and insert into the capsule of first metatarsophalangeal joint. (**D**) Schematic drawing of Subtype 3C. (**E**) Photo of Subtype 3K. Two accessory tendons arise from the lateral side of the main tendon and insert into the capsule of first metatarsophalangeal joint. (**F**) Schematic drawing of Subtype 3K. AT, accessory tendon; EHB, extensor hallucis brevis tendon; EHL, extensor hallucis longus tendon; MT, main tendon.

Type 3 (triple insertions) consists of three tendons including main tendon and two accessory tendon The main tendon inserts as in Type 1; with the following subtypes divided according to the different insertion of the accessory tendons. Type 3 was observed in the 3 feet (1.9%). Subtype 3B and 3C described in the classification by Zielinska et al. were observed in the 1 foot (0.6%), respectively. Subtype 3B showed that two accessory tendons arise from the medial side of the main tendon and insert into the capsule of first metatarsophalangeal joint, whereas two accessory tendons arise from the medial and lateral side of the main tendon and insert into the capsule of first metatarsophalangeal joint in Subtype 3C (Figure 4A–D). However, Subtype 3A was not observed in our study. In addition, it is noteworthy that new subtype not mentioned in the classification by Zielinska et al. was founded in 1 foot (0.6%) of our study. In the subtype named ‘3K’, the two accessory tendons arise from the lateral side of the main tendon and insert into the capsule of first metatarsophalangeal joint (Figure 4E,F).

The significant difference regarding the distribution of types can be found between gender (*p* = 0.016) which is presented in Table 1. However, there was no significant difference in the distribution of types between the left and right (*p* = 0.205) (Table 2).

The length and width of the main tendon of EHL were measured in 158 feet, while the measurement of the length and width of the accessory tendon of EHL were evaluated in 106 feet of Type 2. The mean values of these measurements are presented in Table 3. These measurements did not differ significantly between genders.

## 4. Discussion

Although recently updated classification of the EHL tendon variation to clarify presentation of morphologic characteristics of the accessory tendon of the EHL has been proposed, the contribution of current study is to suggest morphological characteristics of the EHL tendon variation in Korean population, while complementing the previous classification system by observing new types of variation. Additionally, by emphasizing the high morphological variability of the EHL tendons, the current study reinforces the possibility of differences in the morphological variability by race or ethnicity and gender.

In the literature review of incidence rates of an accessory tendon of the EHL, the incidence rate of an accessory tendon of the EHL is relatively low in Saudi Arabia (35.0%) [3], Greece (26.5%) [8], and Poland (42.5%) [9]; whereas in Turkey (78.4%) [5] and the United States (75%) [4], the incidence is high. In particular, two recent studies of the Asian population [6,7] reported that 47 out of 48 feet (97.92%) of a Taiwanese sample and all 50 feet of a Chinese sample had accessory tendons of EHL. The current study was conducted on the largest number of samples to date (158 feet), and the incidence rate of the accessory tendons of EHL was 67.1%. This may mean a high incidence of the accessory tendons of EHL in Asian populations, but it also means that there is high variability in frequency according to region and ethnicity in same Asian populations. Furthermore, a noteworthy point in comparing the frequency of the accessory tendons of EHLs among studies is that there is a wide range of frequencies among adults, from 26.5% to 100% (Table 4). This clearly reinforces a difference in the morphology of the EHL tendon variation by region and race or ethnicity.

A classification system is necessary to clarify the morphology of the EHL tendon variations, and Al-Saggaf [3] created the first accurate classification. This systematic classification was based on the number and the location of insertion of accessory tendons of the EHL. Additionally, Olewnik et al. [9] suggested a systematic classification based on the same criteria but added new subtypes different from the previous one. Most recently, Zielinska et al. [11] proposed a new, detailed classification system which is merely a combination of subtypes from the previous two classifications. In the current study, a subtype in which the medial accessory EHL tendon is inserted into the first interphalangeal joint capsule near the attachment site of the main EHL tendon was observed in 11 cases (7%) and this pattern could be suggested as a new subtype (named ‘Type 2K’). In addition, another new subtype (named ‘Type 3K’) in which two accessory EHL tendons run laterally relative to the major EHL tendon was also suggested (Table 3). Therefore, it is clear that the morphology of the EHL tendon is highly variable. For this reason, the current study suggests a newly updated classification by supplementing the subtypes not presented in the recently updated classification system through dissection of the larger samples.

In addition, in our study, cadaveric dissections were made for 158 cases—a very generous sample size—allowing paired analysis of the feet of each of 79 patients. A variant of the EHL tendon could be obtained with the result that even one patient could show different morphologies between their two sides. These results are noteworthy as they imply that variability of the EHL tendon can occur even within the same individual.

Since the EHL tendon variants are mainly attached to the metatarsophalangeal and interphalangeal joints, hallux deformity—such as hallux valgus—may be developed secondary to imbalance with the action of other tendons around the joint of the big toe. In the literature review, the relationship between EHL tendon variation and hallux valgus is controversial. Many researchers have failed to find any significant correlation between the presence of an accessary EHL tendon and that of hallux valgus [4,5,8]. Natsis et al. [8] even mentioned that the action of the accessory EHL tendon against the axis of deformity force in the varus direction might rather prevent the deformity. Conversely, some authors have thought that an accessory tendon of EHL may influence the development of hallux valgus [3,12]. Our results showed that the frequency of the accessory EHL tendon was significantly higher among females. As a result of a systematic review and meta-analysis of the prevalence of hallux valgus [13], it is clear that hallux valgus is prevalent in women. Therefore, the contribution of the accessory tendon of EHL related to HV deformity seems to remain unclear, and a biomechanical study will be required to determine what direction of force the accessary EHL tendon acts on the hallux according to the classification system.

It has been suggested that the accessory EHL tendon could be used as tendon graft in various reconstructive surgeries such as tendon repairs, small ligament transfers, and interpositional arthroplasty [5,10]. However, its usefulness as donor for tendon graft would depend on its width and length, although tendon length may be less important than width and volume with respect to autogenous grafting. Boyd et al. [14] found that the average length of an accessory EHL tendon in Type 2 was 10.8 cm; and in our results, it was 9.44 cm. Additionally, Boyd et al. [14] suggested that 12 out of 81 feet (14%), when the accessory EHL tendon had a width of 2 mm or more, that it could be used as a donor for tendon graft. Similarly, our results showed that 16 out of 106 feet (15%) had a width of 2 mm or more and the average of the accessory EHL tendon was 1.39 mm. It is considered necessary to check the tendon width using imaging tests such as magnetic resonance imaging (MRI) or ultrasound before surgery in order to determine suitability for grafting, and the newly updated classification of the EHL tendon variation may be helpful to foot and ankle surgeons.

There were some limitations in this study. Fixed cadavers were used to evaluate tendon structure, so there may be differences comparing a live person due to post-mortem changes. Furthermore, as the mean age of cadavers was 79.9 years, the presence of tendon pathology may not be completely ruled out, because the past medical histories of the corpse specimens donated for research are uncertain.

## 5. Conclusions

The present study suggests morphological characteristics of the EHL tendon variation in Korean populations and high morphological variability of the EHL tendon with the possibility of differences according to race or ethnicity and gender. Additionally, a newly updated classification complemented by new subtypes of variation will help foot and ankle surgeons with diagnosis and surgical planning concerning hallux problems.

## Figures and Tables

**Table 1:** Distribution of types according to the gender (*n* = 158).

		Gender			*p*-Value
Type	Subtype	Male	Female	Total	
1		39 (39.0)	10 (17.2)	49 (31.0)	0.016
2		59 (59.0)	47 (81.0)	106 (67.1)	
	A	52 (52.0)	42 (72.4)	94 (59.5)	
	C	0	1 (1.7)	1 (0.6)	
	K	7 (7.0)	4 (6.9)	11 (7.0%)	
3		2 (2.0)	1 (1.8)	3 (1.9)	
	B	1 (1.0)	0	1 (0.6)	
	C	1 (1.0)	0	1 (0.6)	
	K	0	1 (1.8)	1 (0.6)	
Sum		100 (100.0)	58 (100.0)	158 (100.0)	

The data are presented as number (percent).

**Table 2:** Distribution of types according to the side (*n* = 158).

	Side			*p*-Value
Type	Right	Left	Total	
1	26 (33.0)	22 (27.8)	48 (30.3)	0.205
2	50 (63.2)	57 (72.2)	107 (67.7)	
3	3 (3.8)	0 (0)	3 (1.9)	
Sum	79 (100.0)	79 (100.0)	158 (100.0)	

The data are presented as number (percent).

**Table 3:** Length and width of the extensor hallucis longus (EHL) tendon variations.

Measurement	Male	Female	*p*-Value
MT length	151.25 ± 22.23	147.57 ± 26.97	0.266
MT width	54.27 ± 1.55	49.02 ± 1.11	0.111
AT length ^a^	97.35 ± 36.43	90.89 ± 27.53	0.301
AT width ^a^	14.65 ± 6.81	13.06 ± 5.57	0.201

The data (in mm) are presented as mean ± standard deviation. AT, accessory tendon; MT, main tendon. ^a^ Value of the accessory tendon was from Type 2 (*n* = 106 feet).

**Table 4:** Comparison between the current study and previous studies of the extensor hallucis longus (EHL) tendon variations using the classification system by Zielinska et al. [11].

Authors; Year	Denk et al.; 2002 [5]	Al-Saggaf; 2003 [3]	Bibbi et al.; 2004 [4]	Natsis et al.; 2017 [8]	Olewnik et al.; 2019 [9]	Lai et al.; 2021 [6]	Li et al.; 2021 [7]	Current Study
Country	Turkey	Saudi Arabia	United States	Greece	Poland	Taiwan	China	Republic of Korea
Number of feet	63	60	32	98	104	48	50	158
Type 1	21.6%	65.0%	25.0%	73.5%	57.5%	2.08%	0%	31%
Type 2	78.4%	26.6%	75.0%	26.5%	40.5%	93.75%	98%	67.1%
2A	78.4%	3.3%	75.0%	26.5%	29.9%		96%	59.5%
2B		15.0%			4.8%			
2C					5.7%			0.6%
2D		5.0%						
2E		3.3%						
2K							2%	7.0%
Type 3		8.3%			1.9%	4.17	2%	1.9%
3A					1.9%			
3B		5%						0.6%
3C		3.3%					2%	0.6%
3K								0.6%

## Data Availability

The data used to support the findings of this study are available from the corresponding author upon request.
